# Plant Oils Were Associated with Low Prevalence of Impaired Glucose Metabolism in Japanese Workers

**DOI:** 10.1371/journal.pone.0064758

**Published:** 2013-05-31

**Authors:** Kayo Kurotani, Takeshi Kochi, Akiko Nanri, Hiroko Tsuruoka, Keisuke Kuwahara, Ngoc Minh Pham, Isamu Kabe, Tetsuya Mizoue

**Affiliations:** 1 Department of Epidemiology and Prevention, Clinical Research Center, National Center for Global Health and Medicine, Tokyo, Japan; 2 Department of Health Administration, Furukawa Electric Corporation, Tokyo, Japan; University College London, United Kingdom

## Abstract

Fatty acid has been suggested to be involved in development of diabetes. However, its association is unclear among Japanese populations, which consume large amounts of fish rich in n-3 polyunsaturated fatty acids. The present cross-sectional study examined the association of individual dietary fatty acids and dietary fatty acid patterns with abnormal glucose metabolism among 1065 Japanese employees, aged 18–69 years. Impaired glucose metabolism is defined if a person has a history of diabetes, current use of anti-diabetic drug, fasting plasma glucose of 110 mg/dl (≥6.1 mmol/L) or greater, or hemoglobin A1C of 6.0% (≥42 mmol/mol) or greater. Dietary intake was assessed with a self-administered diet history questionnaire. Dietary fatty acid patterns were extracted by principal component analysis. Odds ratios of impaired glucose metabolism according to tertile categories of each fatty acids and dietary fatty acid patterns were estimated using logistic regression with adjustment for potential confounding variables. A higher intake of polyunsaturated fatty acid, n-6 fatty acid, linoleic acid, and oleic acid were significantly associated with a decreased prevalence of impaired glucose metabolism (P for trend = 0.03, 0.01, 0.02, and 0.04, respectively). Alpha-linolenic acid was marginally significantly associated with a decreased prevalence of impaired glucose metabolism (P for trend = 0.12). Of three fatty acid patterns identified, a higher plant oil pattern score, which characterized by high intake of alpha-linolenic acid, linoleic acid, and oleic acid, was associated with a decreased prevalence of impaired glucose metabolism (P for trend = 0.03). No association was observed for other patterns. In conclusion, plant source fatty acids might be protectively associated with development of diabetes in Japanese adults.

## Introduction

Experimental and mechanistic studies have suggested a role of dietary fatty acid composition in glucose metabolism. The dietary fatty acid composition might affect cellular functions such as translocation of glucose transporters and insulin signaling [1]. Indeed, long chain polyunsaturated fatty acids (PUFAs) have been postulated to improve insulin sensitivity [2] and saturated fatty acids impair insulin sensitivity [3] by regulating the cell membrane composition of fatty acids [4]. In addition, recent experimental data showed that dietary fatty acids involve direct regulatory effects on lipogenic gene expression and enzyme activity [5].

Observational studies showed that saturated fatty acid intake was positively associated with insulin resistance and type 2 diabetes, whereas the evidence regarding monounsaturated fatty acid (MUFA) and PUFA were inconclusive [6]. A recent meta-analysis of prospective studies showed no clear association of eicosapentaenoic acid (EPA) and docosahexaenoic acid (DHA) from seafood, whereas EPA and DHA were associated with lower risk of diabetes in Asians studies and with higher risk of diabetes in Western studies [7]. The above meta-analysis showed that a weak inverse association of alpha-linolenic acid (ALA)from plant sources with type 2 diabetes [7]. Regarding linoleic acid (LA), the main dietary n-6 PUFA, an inverse association was generally observed between LA and type 2 diabetes [8]. An intervention study reported a diet rich in n-6 PUFA improved insulin sensitivity compared with a diet rich in saturated fatty acid [9].

To our knowledge, most previous studies on this issue were conducted among Western populations, which have a relatively high body mass [10], higher beta-cell function [11], and lower intake of fish [12], a rich source of long chain n-3 fatty acids, compared with Japanese population. Dietary fatty acid consumption and its effect on glucose metabolism in Japanese population may differ from those in Western populations. In addition, intakes of individual fatty acids are highly inter-correlated and thus it makes difficult to separate their specific effects. Although the examination of dietary fatty acid patterns is important, the relationship between patterns of dietary fatty acid composition and glucose metabolism has not been investigated. We hypothesized that high levels of saturated fatty acids and low levels of both n-6 and n-3 PUFAs and MUFA are associated with abnormal glucose metabolism. To test this hypothesis, we examined cross-sectionally the association of individual dietary fatty acid and patterns of fatty acid intake with diabetes, pre-diabetes, and insulin resistance and secretion in a Japanese working population.

## Materials and Methods

### Study Procedure

In April 2012, a nutritional epidemiological survey was conducted at the time of the periodic health examination among workers of a manufacturing company and its affiliated ones in Chiba Prefecture, Japan. The primary objective of the study was to investigate the association of diet with physical and mental health. Prior to the health check-up, all full-time workers (n = 1,675) were asked to participate in the survey and fill out two types of survey questionnaire (one specifically designed for diet and another for overall health-related lifestyle). Of these, 1,218 (1,076 men and 142 women aged 18–70 years) agreed to participate (response rate, 73%). On the day of health check-up, research staff checked the questionnaire for completeness and, where necessary, clarified with the subjects. Participants were asked to donate 7 mL of venous blood for study. Additionally, we obtained health checkup data including results of anthropometric and biochemical measurements and information on history of disease. The study protocol was approved by the Ethics Committee of the National Center for Global Health and Medicine, Japan. Written informed consent was obtained from all subjects prior to the survey.

### Subjects

Six participants received the health checkup outside the survey period and thus their checkup data were not available. Of the remaining 1,212 participants with health checkup data, we excluded 37 participants who reported a history of cancer (n = 14), cardiovascular disease (n = 14), chronic hepatitis (n = 1), chronic kidney disease including nephritis (n = 8), and pancreatitis (n = 2). We further excluded 88 individuals who did not return dietary questionnaire (n = 6), did not donate blood sample (n = 76), received the examination in non-fasting status (n = 82). Some participants had two or more conditions for exclusion. Of the remaining subjects, we excluded those with missing data on covariates (n = 11). Finally, we excluded 11 subjects with extremely high or low energy intake (exceeding 3 standard deviation), leaving 1065 (953 men and 112 women) for analysis.

### Laboratory Measurement

As part of health checkup, plasma glucose concentration was assayed enzymatically using Quick-auto-neo-GLU-HK (Shino-Test Corp., Tokyo, Japan) and hemoglobin A1c was measured with a latex agglutination immunoassay using the Determiner HbA1c kit (Kyowa Medex Co., Ltd., Tokyo, Japan) at an external laboratory (Kinki Kenko Kanri Center, Shiga, Japan). Venous blood that was donated specifically for the study was drawn into a vacuum tube (7 mL) and then shipped in a cooler box to another laboratory (Mitsubishi Chemical Medience Corporation, Tokyo, Japan), where the blood was centrifuged and serum insulin was measured; the remaining serum sample was stored at –80°C until analysis. Insulin was determined with chemiluminescence immunoassay, with the intra-assay coefficients of variation being 2.5% at 43.1 pmol/l and 1.2% at 423 pmol/l. We computed the homeostasis model assessment-insulin resistance (HOMA-IR) and homeostasis model assessment for β cell function (HOMA-β) using the following formula: HOMA-IR = fasting insulin (µU/ml)×fasting glucose (mmol/ml)/22.5 [HOMA-IR = fasting insulin (µU/ml)×fasting glucose (mg/dl)/405] [13] and HOMA-β = 360×fasting insulin (µU/ml)/(fasting glucose (mg/dl)-63) [HOMA-β = 20×fasting insulin (U/ml)/(fasting glucose (mmol/ml)–3.5)]. [13].

### Definition of Outcome

Impaired glucose metabolism is defined if a person has a history of diabetes, current use of anti-diabetic drug, fasting plasma glucose of 110 mg/dl (≥6.1 mmol/L) or greater, or hemoglobin A1C of 6.0% (≥42 mmol/mol) or greater. Diabetes is defined if a person has a history of diabetes, current use of anti-diabetic drug, fasting plasma glucose of 126 mg/dl (≥7.0 mmol/L) or greater, or hemoglobin A1C of 6.5% (≥48 mmol/mol) or greater. Pre-diabetes is defined if a person without a history of diabetes and current use of anti-diabetic drug has a fasting plasma glucose of 110 to 125 mg/dl (6.1–6.9 mmol/L) or a hemoglobin A1C of 6.0 to 6.4% (42–46 mmol/mol) [14].

### Dietary Assessment

Dietary habit during the preceding month was assessed using a validated brief self-administered diet history questionnaire (BDHQ), which consists of five sections: 1) the frequency of 46 food and non-alcoholic beverage intake; 2) daily frequency of rice and miso soup intake; 3) the frequency of alcoholic drinking and the amount of consumption for five alcoholic beverages per typical drinking occasion; 4) usual cooking method; and 5) dietary behavior [15]. Dietary intakes for 58 food and beverage items, energy, and selected nutrients were estimated using an ad hoc computer algorithm for the BDHQ, with reference to the standard tables of food composition in Japan. According to the validation study of the BDHQ using 16-day weighted dietary records as the gold standard, Pearson correlation coefficient for energy-adjusted intake of saturated fat, monounsaturated fat, and polyunsaturated fat was 0.58 and 0.64, 0.61 and 0.61,and 0.48 and 0.41 in men and women, respectively [16].

We also performed principal component analysis on the basis of energy-adjusted intakes using a density method of 15 fatty acids to derive dietary fatty acid patterns. The factors were rotated by orthogonal transformation (varimax rotation) to maintain uncorrelated factors and greater interpretability. We considered eigenvalues, the scree test and the interpretability of the factors to determine the number of factors to retain. The factors satisfied the criteria for eigenvalues greater than one, and the scree plots dropped substantially. We decided to retain three factors. Dietary fatty acid patterns were named according to fatty acids showing high loading (absolute value) on three factors. The factor scores of each fatty acid pattern for each individual were calculated by summing intakes of fatty acids weighted by their factor loadings.

### Other Variables

Living status, night and rotating shift work, smoking, alcohol drinking, occupational and non-occupational physical activities, and parental history of disease were elicited in the survey questionnaire. Smoking status (never, past, or current) and, if past or current smoker, duration of smoking in years and numbers of cigarettes smoked per day were asked. Averaged daily ethanol intake from alcohol beverage was calculated as drinking frequency multiplied by ethanol consumption per drinking day. Regarding physical activity, participants were asked about work-related activity (including domestic housework and commuting to and from work) and leisure-time activity. Work-related and leisure-time activities were each expressed as the sum of metabolic equivalent (MET) value multiplied by the duration of the activity within each domain. Body height was measured to the nearest 0.1 cm with subjects standing without shoes. Body weight in light clothes was measured to the nearest 0.1 kg. Body mass index (BMI) was calculated as weight in kilograms divided by squared height in meters.

### Statistical Analysis

Participants were divided into ‘tertiles’ according to total fat intake (% energy). Data were expressed as means (SD) and percentages for continuous variables and categorical variables, respectively, adjusted for age and sex. Their differences across tertiles categories were tested using linear regression analysis or multiple logistic regression for trend, for continuous and categorical variables, respectively. Logistic regression analysis was performed to estimate (prevalence) odds ratio and its 95% confidence interval for impaired glucose metabolism according to tertile of each fatty acid or factor scores, and the lowest tertile was used as the reference category. Trend association was assessed by assigning ordinal numbers to each tertile of each fatty acid. Model 1 was adjusted for age (continuous, yr) and sex only, and Model 2 was additionally adjusted for night or rotating shift work (yes or no), parental history of diabetes, BMI (continuous, kg/m^2^), smoking (never-smoker, quitter, current smoker consuming <20 cigarettes per day, or current smoker consuming ≥20 cigarettes per day), alcohol drinking (nondrinker including infrequent drinker consuming less than once per week, drinkers consuming <23 g per day, drinkers consuming ≥23 to <46 g per day, or drinkers consuming ≥46 g per day), work-related physical activity (<3 MET-h/d, 3–<7 MET-h/d, 7–<20 MET-h/d, or ≥20 MET-h/d), leisure-time physical activity (0 MET-h/week, 1<–<3 MET-h/week, 3–<10 MET-h/week, or ≥10 MET-h/week), anti-hypertensive treatment (yes or no), anti-dyslipidemia treatment (yes or no), energy intake (continuous in log-scale, kcal per day), protein intake (continuous, % energy). We also analyzed diabetes (n = 61) and pre-diabetes (n = 58) separately. Logarithmic transformation of HOMA-IR and HOMA-beta was made before analysis and we excluded 54 subjects due to missing data on insulin. Multiple linear regression was used to calculate mean and its 95% confidence interval of HOMA-IR and HOMA-beta across tertile of dietary fatty acid scores. Two-sided *P* values of less than 0.05 were considered as statistically significant. All analyses were performed using Statistical Analysis System (SAS) software version 9.1 (SAS Institute, Cary, NC, USA).

## Results

Characteristics according to tertile categories of % energy of fat intake are shown in Table 1. Participants with a higher total fat intake were more likely to be young and female and to consume protein and less likely to consume carbohydrate, and to be physically active in job, day shift workers, current smoker, and current alcohol drinker.

**Table 1:** Age and sex- adjusted characteristics of participants according to quartile categories of total fatty acid intake (Ene %).

	Tertile categories of total fatty acid intake (Ene %)			
	Tertile 1 (low)	Tertile 2	Tertile 3 (high)	P^1^
Median (Ene %)	18.7	23.4	28.6	
No. of subjects	355	355	355	
Sex (male, %)	95.2	92.4	80.9	<.0001
Age (year)^2^	45.4±9.5	44.2±9.5	43.9±9.6	0.04
BMI (kg/m^2^)^3^	23.3±3.3	23.2±3.3	23.4±3.4	0.83
Work rotation (day shift, %)^4^	25.5	17.7	11.6	<.0001
Work-related physical activity (≥20 Mets-h/d, %)^4^	27.9	24.0	18.6	0.004
Leisure time physical activity (≥10 Mets-h/w, %)^4^	23.4	28.7	20.8	0.44
Smoking status (current, %)^4^	32.5	23.7	20.4	<.0001
Alcohol consumption (≥1 d/w, %)^4^	35.4	25.6	16.1	<.0001
Parental history of diabetes (yes, %)^4^	14.1	14.9	16.9	0.47
Hypertension (yes, %)^4^	10.8	7.8	8.4	0.23
Hyperlipidemia (yes, %)^4^	5.7	4.3	5.8	0.99
Total energy intake (kcal/d) 3	1802±482	1861±479	1811±485	0.79
Carbohydrate (% energy) 3	58.8±7.2	55.8±7.2	50.5±7.3	<.0001
Protein (% energy) 3	12.2±2.1	14.0±2.1	15.6±2.1	<.0001

1 Based on logistic regression for categorical variables and linear regression analysis for continuous variables, assigning ordinal numbers 0–2 to tertile categories of total fatty acid intake.

2 Sex adjusted means±SD.

3 Age and sex adjusted means±SD.

4 Age and sex adjusted proportions.

In the present study, we identified 61 diabetes and 58 pre-diabetes participants. Table 2 shows the odds ratios of impaired glucose metabolism according to tertile categories of each fatty acid intake (% energy). Multiple logistic regression showed that the prevalence of impaired glucose metabolism was decreased as increased PUFA (P for trend = 0.03), n-6 PUFA (P for trend = 0.01), oleic acid (18∶1) (P for trend = 0.04), and LA (18∶2 n-6) (P for trend = 0.02). Although ALA (18∶3 n-3) was non-significantly inversely associated with impaired glucose metabolism odds in multivariable model (P for trend = 0.12), there were no significant associations between impaired glucose metabolism and marine n-3 fatty acids. Saturated fatty acids were also not associated with impaired glucose metabolism. In the analysis for diabetes, a higher intake of n-6 PUFA was significantly inversely associated with odds of diabetes; the multivariate-adjusted OR (95% CI) of diabetes for the lowest through highest tertile of PUFA intake were 1.00 (referent), 0.49 (0.22–1.05), 0.40 (0.18–0.91) (P for trend = 0.03). LA was significantly inversely associated with diabetes odds in multivariable model (P for trend = 0.04). In the analysis for pre-diabetes, multiple logistic regression showed that the prevalence of pre-diabetes was decreased with increasing levels of PUFA (P for trend = 0.04).

**Table 2:** The odds ratio (OR) of impaired glucose metabolism (n = 119) according to tertile categories of each fatty acid intake (% energy).

	Median(% energy)	Model 1^1^				Model 2^2^			
		T1	T2	T3	P_trend_ 3	T1	T2	T3	P_trend_ 3
SFA	5.86	1.00 (reference)	1.29 (0.81–2.05)	0.94 (0.56–1.57)	0.89	1.00 (reference)	1.27 (0.73−2.21)	0.80 (0.41−1.55)	0.55
MUFA	8.46	1.00 (reference)	0.92 (0.57−1.49)	1.09 (0.67−1.78)	0.75	1.00 (reference)	0.81 (0.47−1.41)	0.57 (0.30−1.06)	0.08
**PUFA**	6.01	1.00 (reference)	0.89 (0.55−1.45)	1.12 (0.69−1.81)	0.66	1.00 (reference)	**0.64 (0.36**−**1.13)**	**0.48 (0.25**−**0.93)**	**0.03**
n3 PUFA	1.14	1.00 (reference)	1.11 (0.65−1.88)	1.78 (1.09−2.91)	0.02	1.00 (reference)	0.82 (0.44−1.53)	1.00 (0.50−2.01)	0.96
**n6 PUFA**	4.80	1.00 (reference)	0.86 (0.53−1.38)	0.98 (0.60−1.60)	0.91	1.00 (reference)	**0.62 (0.35**−**1.07)**	**0.45 (0.24**−**0.84)**	**0.01**
16∶0	3.58	1.00 (reference)	1.30 (0.81−2.07)	1.06 (0.63−1.77)	0.76	1.00 (reference)	1.37 (0.78−2.40)	0.81 (0.41−1.58)	0.57
16∶1	0.34	1.00 (reference)	1.37 (0.82−2.26)	1.75 (1.06−2.88)	0.03	1.00 (reference)	0.88 (0.48−1.61)	1.15 (0.55−2.39)	0.72
18∶0	1.29	1.00 (reference)	1.27 (0.80−2.03)	1.01 (0.60−1.70)	0.88	1.00 (reference)	1.26 (0.73−2.20)	0.67 (0.35−1.30)	0.27
**18∶1**	7.66	1.00 (reference)	0.90 (0.56−1.45)	0.99 (0.61−1.63)	0.95	1.00 (reference)	**0.85 (0.49**−**1.45)**	**0.51 (0.28**−**0.96)**	**0.04**
**18∶2 n6**	4.69	1.00 (reference)	0.93 (0.58−1.50)	1.00 (0.61−1.63)	0.98	1.00 (reference)	**0.68 (0.39**−**1.18)**	**0.46 (0.25**−**0.87)**	**0.02**
18∶3 n3	0.73	1.00 (reference)	1.14 (0.70−1.86)	1.17 (0.72−1.90)	0.53	1.00 (reference)	1.03 (0.60−1.79)	0.62 (0.34−1.13)	0.12
18∶3 n6	0.003	1.00 (reference)	1.25 (0.76−2.07)	1.39 (0.86−2.27)	0.19	1.00 (reference)	1.20 (0.68−2.13)	1.24 (0.69−2.26)	0.48
18∶4 n3	0.03	1.00 (reference)	0.84 (0.49−1.45)	1.64 (1.00−2.68)	0.03	1.00 (reference)	0.84 (0.46−1.56)	1.52 (0.79−2.94)	0.19
20∶2 n6	0.02	1.00 (reference)	1.64 (0.99−2.71)	1.73 (1.04−2.86)	0.03	1.00 (reference)	1.37 (0.76−2.47)	1.03 (0.54−1.99)	0.97
20∶3 n6	0.01	1.00 (reference)	1.35 (0.82−2.22)	1.56 (0.95−2.57)	0.08	1.00 (reference)	1.19 (0.66−2.14)	1.08 (0.54−2.16)	0.83
20∶4 n6	0.07	1.00 (reference)	1.53 (0.93−2.53)	1.55 (0.94−2.58)	0.09	1.00 (reference)	1.20 (0.66−2.18)	0.98 (0.49−1.98)	0.92
20∶5 n3	0.11	1.00 (reference)	1.06 (0.62−1.81)	1.68 (1.02−2.76)	0.03	1.00 (reference)	0.96 (0.52−1.77)	1.38 (0.71−2.69)	0.32
22∶5 n3	0.03	1.00 (reference)	1.30 (0.76−2.23)	1.92 (1.15−3.20)	0.01	1.00 (reference)	1.12 (0.60−2.10)	1.50 (0.75−3.01)	0.24
22∶5 n6	0.003	1.00 (reference)	0.93 (0.55−1.55)	1.33 (0.82−2.15)	0.23	1.00 (reference)	0.78 (0.42−1.44)	1.06 (0.55−2.02)	0.84
22∶6 n3	0.20	1.00 (reference)	1.16 (0.68−1.99)	1.77 (1.07−2.92)	0.02	1.00 (reference)	1.09 (0.59−2.01)	1.39 (0.70−2.77)	0.34

1 Adjusted for age (y) and sex.

2 Adjusted for age (y) sex, BMI (kg/m^2^), shiftwork (yes or no), leisure time physical activity (0, 1<–<3, 3–<10, ≥10 Mets-h/w), work-related physical activity (<3, 3–<7, 7–<20, ≥20 Mets-h/w), smoking status (never and past, current and <20 cigarette/d, or current and ≥20 cigarette/d), alcohol consumption (nondrinker and 1–3 d/m, <23 g ethanol/d, 23–<46 g ethanol/d, or ≥46 g ethanol/d), hypertension (yes or no), hyperlipidemia (yes or no), parental history of diabetes (yes, no or unknown), log transformed total energy intake (kcal/d), and protein intake (% energy).

3 Based on multiple linear regression analysis, assigning ordinal numbers 0−2 to tertile categories of each fatty acid intake.

Additionally, we examined the association of individual fatty acids with HOMA-IR and HOMA-beta. There were no significant associations of any fatty acids with HOMA-IR and HOMA-beta (data not shown). However, gamma-linolenic acid (GLA) (18∶3 n−6) and EPA (20∶5 n−3) were marginally significantly positively associated with HOMA-beta; geometric means (95% CI) for the tertile categories from the lowest to the highest were 59.0 (55.9−62.4), 60.9 (57.8−64.2), and 63.6 (60.2−67.2) (P for trend = 0.07) for GLA and 58.6 (55.3−62.1), 61.3 (58.2−64.6), and 63.8 (60.0−67.7) (P for trend = 0.07) for EPA.

We identified three dietary fatty acid patterns (Table 3). The first pattern was characterized by high intakes of 20∶5 n-3, 18∶4 n-3, 22∶5 n-3, and 22∶6 n-3, and thus was named a fish oil pattern. The second pattern represented high intakes of 20∶3 n-6, 18∶0, and 16∶0, and thus it was called a meat oriented pattern. The third pattern was characterized by high intakes of 18∶3 n-3, 18∶2 n-6, and 18∶1. These fatty acids are derived from plant souses, and this pattern was named as a plant oil pattern.

**Table 3:** Factor loading matrix for the major fatty acid patterns identified by principal component analysis.

	Fish oil pattern	Meat oriented pattern	Plant oil pattern
20∶5 n-3	0.98	0.09	0.03
18∶4 n-3	0.98	0.10	0.04
22∶5 n-3	0.97	0.17	0.04
22∶6 n-3	0.97	0.16	0.05
22∶5 n-6	0.93	0.18	0.04
18∶3 n-6	0.58	0.32	−0.14
20∶3 n-6	0.29	0.92	0.12
18∶0	−0.02	0.92	0.32
16∶0	0.05	0.91	0.29
20∶2 n-6	0.25	0.87	0.21
16∶1	0.50	0.78	0.21
20∶4 n-6	0.38	0.76	0.12
18∶1	−0.04	0.73	0.65
18∶3 n-3	0.05	0.26	0.95
18∶2 n-6	0.002	0.38	0.90
Variance explained (%)	54.1	27.4	8.1

Of the three dietary patterns, the plant oil pattern showed a significant inverse association with prevalence of impaired glucose metabolism (Table 4). Multivariate-adjusted OR (95% CI) for the tertile categories (from the lowest to the highest) were 1.00 (referent), 0.80 (0.47−1.37), 0.54 (0.30−0.95) (P for trend = 0.03). In the analysis for diabetes, higher scores of the plant oil pattern was significantly inversely associated with odds of diabetes; the multivariate-adjusted OR (95% CI) of diabetes for the lowest through highest tertile of plant oil pattern score were 1.00 (referent), 0.57 (0.27−1.19), 0.46 (0.22−0.96) (P for trend = 0.04). However, in the analysis for pre-diabetes, the inverse association with plant oil pattern score was attenuated; the corresponding values for the lowest through highest tertile of plant oil pattern score were 1.00 (referent), 1.36 (0.66−2.79), 0.61 (0.27−1.38) (P for trend = 0.28). Neither fish oil pattern nor meat oriented pattern was associated with prevalence of impaired glucose metabolism. As regards to HOMA-IR and HOMA-beta, there were no significant associations with scores of any dietary fatty acid patterns.

**Table 4:** The odds ratio (OR) of impaired glucose metabolism (n = 119) according to tertile categories of fatty acid pattern score.

	Fish oil pattern		Meat oriented pattern		Plant oil pattern	
	Model 1^1^	Model 2^2^	Model 1^1^	Model 2^2^	Model 1^1^	Model 2^2^
Impaired glucose metabolism^3^						
Tertile 1 (low)	1.00 (reference)	1.00 (reference)	1.00 (reference)	1.00 (reference)	1.00 (reference)	1.00 (reference)
Tertile 2	1.18 (0.69−2.02)	1.05 (0.57−1.94)	1.43 (0.89−2.30)	1.29 (0.75−2.24)	0.81 (0.49−1.31)	**0.80 (0.47**−**1.37)**
Tertile 3 (high)	1.79(1.07−2.95)	1.47 (0.77−2.82)	1.20 (0.72−2.00)	0.87 (0.46−1.65)	0.93 (0.58−1.50)	**0.54 (0.30**−**0.95)**
P trend^4^	0.02	0.23	0.45	0.71	0.76	**0.03**
Diabetes^5^						
Tertile 1 (low)	1.00 (reference)	1.00 (reference)	1.00 (reference)	1.00 (reference)	1.00 (reference)	1.00 (reference)
Tertile 2	0.98 (0.50−1.96)	0.94 (0.43−2.05)	2.13 (1.11−4.10)	1.74 (0.82−3.70)	0.63 (0.32−1.23)	**0.57 (0.27**−**1.19)**
Tertile 3 (high)	1.25 (0.65−2.39)	0.99 (0.42−2.35)	1.52 (0.75−3.10)	0.89 (0.38−2.10)	0.93 (0.51−1.71)	**0.46 (0.22**−**0.96)**
P trend^4^	0.48	0.98	0.23	0.75	0.80	**0.04**
Pre−diabetes^6^						
Tertile 1 (low)	1.00 (reference)	1.00 (reference)	1.00 (reference)	1.00 (reference)	1.00 (reference)	1.00 (reference)
Tertile 2	1.44 (0.63−3.26)	1.19 (0.48−2.93)	0.84 (0.44−1.63)	0.89 (0.42−1.89)	1.04 (0.54−2.00)	1.36 (0.66−2.79)
Tertile 3 (high)	2.53 (1.20−5.35)	2.09 (0.84−5.25)	0.43 (0.47−1.84)	0.95 (0.41−2.21)	0.90 (0.46−1.77)	0.61 (0.27−1.38)
P trend^4^	0.01	0.09	0.80	0.88	0.76	0.28

1 Adjusted for age (y) and sex.

2 Adjusted for age (y) sex, BMI (kg/m^2^), shiftwork (yes or no), leisure time physical activity (0, 1<–<3, 3–<10, ≥10 Mets-h/w), work-related physical activity (<3, 3–<7, 7–<20, ≥20 Mets-h/w), smoking status (never and past, current and <20 cigarette/d, or current and ≥20 cigarette/d), alcohol consumption (nondrinker and 1–3 d/m, <23 g ethanol/d, 23–<46 g ethanol/d, or ≥46 g ethanol/d), hypertension (yes or no), hyperlipidemia (yes or no), parental history of diabetes (yes, no or unknown), log transformed total energy intake (kcal/d), and protein intake (% energy).

3 Number of cases of tertile 1 to tertile 3 was 27, 35, and 57 for factor 1, 40, 45, and 34 for factor 2, and 44, 35, and 40 for factor 3, respectively.

4 Based on multiple linear regression analysis, assigning ordinal numbers 0−2 to tertile categories of each fatty acid intake.

5 Number of cases of tertile 1 to tertile 3 was 17, 18, and 26 for factor 1, 16, 27, and 18 for factor 2, and 24, 15, and 22 for factor 3, respectively.

6 Number of cases of tertile 1 to tertile 3 was 10, 16, and 32 for factor 1, 25, 17, and 16 for factor 2, and 20, 20, and 18 for factor 3, respectively.

## Discussion

In this cross-sectional study of a Japanese working population, we found inverse relations of intake of PUFA, n-6 PUFA, oleic acid, and LA with impaired glucose metabolism. ALA was associated with lower prevalence of impaired glucose metabolism. Of three dietary fatty acid patterns identified, i.e. fish oil pattern, meat oriented pattern, and plant oil pattern, we also found an inverse association of impaired glucose metabolism with plant oil pattern, which was characterized by high intake of ALA, LA, and oleic acid. Fatty acids or fatty acid patterns were not associated with markers of insulin resistance and secretion. To our knowledge, this is the first study among Japanese, who consume high amount of fish (long-chain n-3 PUFAs), that investigates dietary fatty acid pattern in relation to glucose metabolism abnormality.

We observed inverse associations of intake of PUFA, n-6 PUFA, and LA (a major contributor to PUFA and n-6 PUFA) with impaired glucose metabolism. Previously, some [17], [18] but not all [19] prospective studies showed an inverse association of risk of type 2 diabetes with intake of PUFA, and an intervention study also reported that a diet rich in LA improved insulin sensitivity compared with saturated fatty acid rich diet [9]. In the Health Professional Follow-up Study, although among overall participants intake of LA was not associated with risk of type 2 diabetes, among men with <65 years of age and with a BMI <25 kg/m^2^ LA was observed a statistically significantly inverse association [20]. The present study population was young (aged 44.5±9.5 years) and lean (proportion of BMI <25 kg/m^2^ = 75%). In addition, several studies have consistently shown an inverse association of LA in serum cholesterol esters or phospholipids with risk of type 2 diabetes [21]–[24]. Taken together, a high intake of PUFA, especially LA, might be protective for glucose intolerance.

We found that oleic acid intake was associated with decreased prevalence of impaired glucose metabolism. Our finding is in line with a cross-sectional study in Italy that showed an inverse association between olive oil, the main source of oleic acid, intake and fasting plasma glucose [25]. Furthermore, both the KANWU study (Sweden), the largest randomized controlled clinical trial substituting dietary SFA for MUFA in healthy men and women, and another controlled short-term trial in healthy subjects in Spain reported that substitution of MUFA for SFA improved insulin sensitivity [26], [27]. However, prospective cohort studies in Western countries showed no clear association of oleic acid or MUFA with risk of diabetes after adjustment for BMI and other covariates [17], [18], [20]. Prospective studies in Asian countries are needed to confirm the association between MUFA and glucose metabolism.

As regards ALA, a plant derived n-3 PUFA, subjects in the highest tertile of intakes had a 38% lower prevalence of both impaired glucose metabolism compared with those in the lowest tertile, although the reductions were not statistically significant. Our result agrees with a meta-analysis of prospective studies that showed a weak inverse association between ALA intake and type 2diabetes [7]. As regards insulin resistance, we found no clear association between ALA and HOMA-IR. In contrast, a Japanese study of middle-aged employees showed that higher ALA intake was significantly associated with a lower prevalence of insulin resistance among normal weight subjects [28]. Although ALA might be related to lower risk of type 2 diabetes, the underlying mechanism should be clarified.

A meta-analysis of 16 cohort studies showed that dietary EPA+DHA were not associated with diabetes (relative risk per 250 mg/d = 1.04, 95% CI = 0.97−1.10) [29]. However, substantial heterogeneity was evident according to geographical regions.; the summary risk estimates indicated no association among European countries, a positive association among US studies, and an inverse association among Asian/Australian studies [29]. We observed no clear associations of marine fatty acids intake with prevalence of impaired glucose metabolism and insulin resistance in a Japanese population. Our finding agreed with that of a cross-sectional study in Japan showing no significant associations of EPA+DHA with insulin resistance [28]. Similarly, a recent meta-analysis of randomized control trials during 2 months to 6 months showed n-3 PUFA had no effects on insulin sensitivity [30]. The effects of n-3 PUFA on glucose metabolism are still debated.

In the present study, plant oil pattern characterized by high intake of LA, ALA, and oleic acid was inversely associated with impaired glucose metabolism. Given that higher levels of these fatty acids were each associated with decreased prevalence of impaired glucose metabolism in this study, the observed association with plant oil pattern would be reasonably expected. In addition, the present finding is consistent with those of previous studies. Hu et al. report that a higher intake of vegetable fat is associated with beneficial glucose metabolism and insulin resistance [31]. Furthermore, the Iowa Women’s Health Study and the Nurses’ Health Study also showed vegetable fat was related to decreased diabetes risk [17], [18] and an Italian cross-sectional study showed that consumption of vegetable oil was inversely associated with serum glucose levels in both sexes [25]. It is possible that plant oil pattern represents the combination of several subtypes of fatty acid that are beneficial for glucose metabolism.

The mechanism underlying the association between fatty acid composition and glucose metabolism is unclear. Fatty acid composition of the adipose cell membrane alters glucose uptake by regulating the translocation and by controlling membrane fluidity involved in glucose transporter 4 [32]. These alteration could affect tissue and whole body insulin sensitivity [33]. Moreover, PUFAs modulate peroxisome proliferator-activated receptor α (PPAR-α) involved in oxidative process and sterol regulatory element-binding proteins (SREBPs) involved in lipogenesis and expression of PPAR-α and SREBPs is decreased with PUFAs [34]. Furthermore, n-6 PUFAs and ALA possibly decrease inflammation through inhibiting the production of inflammatory factors in endothelial cells by down-regulating nuclear factor-kB [35], which can lead to local insulin resistance [36]. However, the present study showed no significant association between these fatty acids and markers of insulin resistance.

Major strengths of this study include a high participation rate, and use of validated BDHQ and the definition of diabetes and pre-diabetes based on multiple sources of information (self-report, HbA1c, and fasting glucose) and adjustment for potentially important confounders. Limitations of our study also warrant mention. First, an association derived from a cross-sectional study does not indicate causality. However, our finding is also supported by those from prospective and intervention studies indicating a favorable role of LA [9], [17], [18], [20] and plant oil [17], [18] in glucose metabolism. In addition, observational studies using serum fatty acid composition, which reflects relatively long term fat intake, have shown an inverse association between LA and impaired glucose metabolism [21]–[24]. Furthermore, we found an inverse association of n-6 PUFA with pre-diabetes, a status which might not influence dietary behavior. We thus believe that the present finding cannot fully be accounted for by reverse causation. Second, dietary information was assessed using a self-administered BDHQ, which is subject to measurement error, probably in a random manner. The resulting misclassification must have distorted the estimate of association toward the null. Third, although we adjusted for potentially important confounding variables, a possibility of residual confounding and unknown confounders cannot be excluded. Finally, because the study participants were workers of a company in Japan, the present findings might not be representative for general population.

In conclusion, high intake of plant source fatty acid, i.e. LA, oleic acid, and ALA was associated with decreased prevalence of impaired glucose metabolism in Japanese men and women, suggesting a favorable role of plant oils in glucose metabolisms. Additional studies are warranted to confirm the role of MUFA and n-3 PUFA involving glucose metabolism in healthy populations using different approaches such as randomized feeding or supplementation studies.
